# Stage Specific Expression Pattern of Alpha-Hemoglobin-Stabilizing-Protein (AHSP) Portrayed in Erythroblast Chronology

**DOI:** 10.3390/mps3030046

**Published:** 2020-06-30

**Authors:** Julia Walczak, Maria D. Camargo Johnson, Kuzhali Muthumalaiappan

**Affiliations:** 1 Burn and Shock Trauma Research Institute, Loyola University Chicago, Health Sciences Division, Maywood, IL 60153, USA; jwalczak@luc.edu (J.W.); mcamargo@luc.edu (M.D.C.J.); 2 Department of Surgery, Loyola University Chicago, Health Sciences Division, Maywood, IL 60153, USA

**Keywords:** AHSP, alpha-hemoglobin-stabilizing-protein, erythropoiesis, enucleation, human bone marrow, biphasic erythroid culture, AHSP-gene, AHSP-flow cytometry, Amnis

## Abstract

During erythropoiesis, the molecular chaperone alpha-hemoglobin-stabilizing protein (AHSP) sequesters free alpha-hemoglobin (*α*Hb) and prevents precipitation of excess *α*Hb. While AHSP is linked to hereditary anemia, the pattern of expression during specific erythroblast stages is poorly understood. We investigated gene and protein expressions of AHSP throughout progressive maturation stages of erythroblasts in biphasic cultures of blood and bone marrow samples from healthy donors. Differentiating erythroblasts were periodically subjected to flow cytometry, Amnis imaging and RT-qPCR analyses. We made parallel in vivo validations from naive murine bone marrow cells. Percentages of AHSP^+^ erythroblasts, protein expressions and AHSP gene expressions are negligible on culture day 6 (CFU-Es) and progressively increases from culture days 8–12 (peaks on day 12) and declines on day 14. Notably, sub-cellular location of AHSP is both in the cytoplasm and nucleus in the early erythroblasts while in the late stages of maturation AHSP is found predominantly in the nucleus, being expelled with it during enucleation. As both human bone marrow and peripheral blood mononuclear cells (PBMC) derived erythroblasts demonstrated similar expression patterns, sampling of erythroblasts from day 11 cultures could portray erythroblast chronology and provide optimum representative stage specific expression patterns. PBMCs may be suitable for comparison studies of AHSP expression in pathologic erythropoiesi

## 1. Introduction

Alpha-hemoglobin-stabilizing protein (AHSP) is a molecular chaperone protein that sequesters free alpha-hemoglobin (*α*Hb). AHSP was first experimentally measured by the Clinton lab in 2001 [1] followed by the seminal report elucidating its functional role in 2002 by the Weiss lab [2]. During the formation of the hemoglobin tetramer, or hemoglobinization, two units of both *α*Hb and beta-hemoglobin (βHb) must coordinate bond with a ferrous iron (Fe) prosthetic group in order to create a functional hemoglobin protein. Unlike βHb, free *α*Hb can neither bind to a heme nor self-associate, but instead aggregates onto the cell membrane. *α*Hb precipitation results in apoptosis of immature erythroid cells and reduced lifespan of erythrocytes [3,4].

Due to the high tissue specificity of hemoglobin (Hb), the body’s primary means of transporting and utilizing oxygen, AHSP is predominantly expressed in hematopoietic tissues and erythroid progenitors [3]. Much of AHSP’s function was studied in hereditary anemia; it has not been considered in other anemia such as anemia of inflammation/critical illness, which is refractory to erythropoietin administration [5,6]. Our lab has found implications of AHSP in burn patients during late stage erythropoiesis (manuscript under review) while investigating peripheral blood mononuclear cells (PBMC) derived erythroblasts in ex vivo cultures [7]. The pattern of AHSP protein expression during erythropoiesis is not fully understood, but gene expression during erythroid maturation is reported by Dos Santos at al. [8]. Thus far, little is known regarding AHSP as it is still a relatively new protein in the field of erythropoiesis. In this methodological study we have portrayed a stage specific expression pattern of AHSP in erythroblast chronology. Results underscore AHSP gene expression, protein expression and localization over its developmental trajectory in ex vivo culturing from human bone marrow cells and human PBMCs. For validation, we have also compared our ex vivo observations with in vivo early and late stage erythroblasts obtained from murine bone marrow.

## 2. Results

### 2.1. AHSP Protein and Gene Expression Peaks on Day 12 During Ex Vivo Erythropoiesis of Bone Marrow Cells

**Figure 1:**
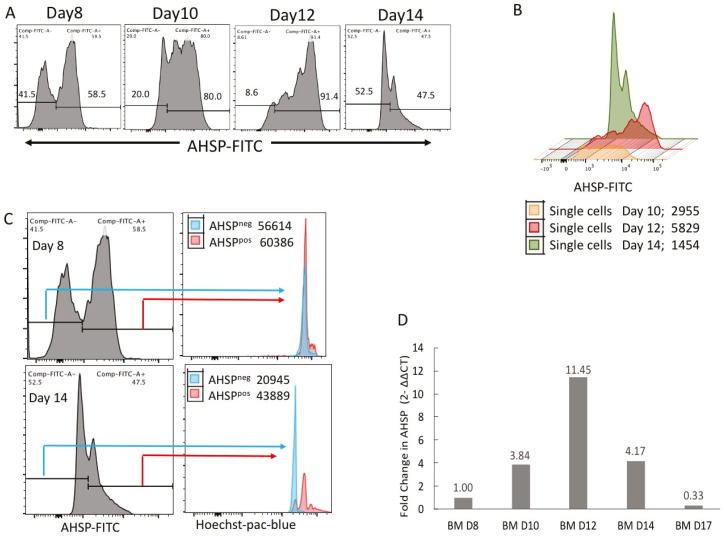
AHSP protein and gene expression in naïve human bone marrow derived erythroblasts. (**A**) Percentage of AHSP positive and negative cells in fluorescence-activated cell sorting (FACS) analysis on culture days 8, 10, 12 and 14. (**B**) Overlay of AHSP protein expression displayed with color graphs on Day 10 (orange), Day 12 (red) and Day 14 (green) showing that the highest mean fluorescent intensity (MFI) shifted to the right on Day 12. (**C**) AHSP positive and negative subsets from culture days 8 and 14 stratified by Hoechst nuclear stain mean fluorescent intensity. (**D**) Relative gene expression of AHSP in total erythroblasts on culture days 8, 10, 12, 14 and 17. Bar graphs represent fold change compared to culture day 8. Target gene cycle threshold (Ct) values were normalized to GAPDH Ct values using the ΔΔCt method.

The histograms in Figure 1A displays the percentage of AHSP positive cells over culture days 8, 10, 12 and 14. When bone marrow cells from a healthy human donor were placed in ex vivo erythropoiesis culture, there was no detectable AHSP measured prior to day 8. Over half of the cells (58.5%) in culture expressed AHSP on day 8, expression increased substantially to 80% on day 10, and peaked at 91.4% on day 12, whereas on day 14 it noticeably decreased to levels less than on day 8–47.5%. Similarly, the amount of AHSP as measured by mean fluorescent intensity (MFI) was the highest on day 12. The overlay of AHSP fluorescence from day 10, day 12 and day 14 cultured cells show day 12 cells fluoresce furthest to the right on the *x* axis logarithmic fluorescence measurement (Figure 1B; Day 10 = 2955, Day 12 = 5829 and Day 14 = 1454).

A defining biological measure of red blood cell maturation is the ejection of the nucleus from developing red blood cells, which began to happen at day 14 of cell culture. On day 8, cells that were both positive and negative for the expression of AHSP had their nucleus present as evidenced from the overlap in the positive Hoechst nuclear stain (Figure 1C; top panel). Distinctly, on day 14 of red blood cell maturation those cells that did not express AHSP also did not stain positively for the presence of their nucleus illustrating they reached maturity (Figure 1C; bottom panel). This implies that ASHP was no longer expressed when erythropoietic cells became reticulocytes devoid of their nuclei. Interestingly, on this same day 14, the cells that express AHSP also stained positively for the presence of their nucleus suggesting they had not yet matured into reticulocytes.

In addition to peak AHSP protein expression on day 12 of ex vivo bone marrow cell culture, AHSP peak mRNA levels coincided with an 11.45-fold increase compared to baseline levels of mRNA at day 8 (Figure 1D). As with AHSP protein expression, there is an increasing AHSP mRNA trend from day 8 to day 12, which is then followed by decreased levels on day 14, at 4.17-fold, and even lower levels on day 17, at 0.33-fold. AHSP gene expression was normalized to the steady gene expression of the housekeeping gene GAPDH.

### 2.2. AHSP Translocation from the Cytoplasm to the Nucleus During Red Blood Cell Maturation

**Figure 2:**
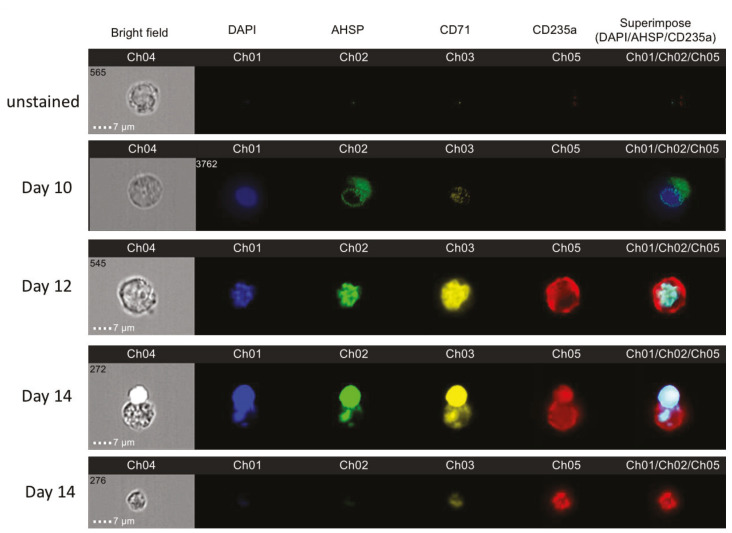
Representative images from AMNIS image flow of bone marrow derived erythroblasts on culture days 10, 12 and 14 with unstained cells for baseline comparison. Cells were examined in all four fluorescent channels and brightfield. Blue stain is the nuclear stain DAPI, green is the intra cellular AHSP marker, yellow is the transferrin marker CD71 and red is the glycophorin A marker CD235a. The superimposed image shows a combination of CD235a, AHSP and DAPI.

We used Amnis imaging, which combines fluorescence-activated cell sorting (FACS) detection of AHSP bound fluorescently labeled antibodies in single cells with photographic imaging capabilities. We were able to uniquely quantify AHSP fluorescent intensities but also physically image the cell and intracellular localization of AHSP throughout various time points in the developmental stages of red blood cell development. The nucleus is identifiable as a spherical shape in the bright field image depending on the position of the cell in relation to the camera and additionally by the Hoechst staining that has been labeled in blue. After qualitatively selecting for images that are in focus, next we gated for cells to exclude debris in our analysis of photographic images. To consistently gate for erythroblasts, as done in FACS analysis, we gated for cells positive for erythroblast markers CD71 (shown in yellow) and CD235a (shown in red). In these gated cells, AHSP appeared in green and could be identified in the cytoplasm but also strongly fluorescing in the nucleus on day 10 (Figure 2). In contrast, AHSP was notably localized solely in the nucleus on day 12 and 14 of ex vivo culturing and no longer present in the cytoplasm. Interestingly, day 14 culturing shows the process of the ejection of the nucleus and AHSP’s ejection along with it. The image captured on day 14 shows some nuclear component still remaining in the cytoplasm suggesting enucleation was still in progress. The bottom-most row depicts a cell in which maturity was attained as illustrated by the absence of nuclear remnants, and also no presence of AHSP further, confirming our flow cytometric observation on Hoechst negativity in AHSP negative cells in Figure 1C.

### 2.3. AHSP Protein and Gene Expression In Vivo Mouse Bone Marrow

**Figure 3:**
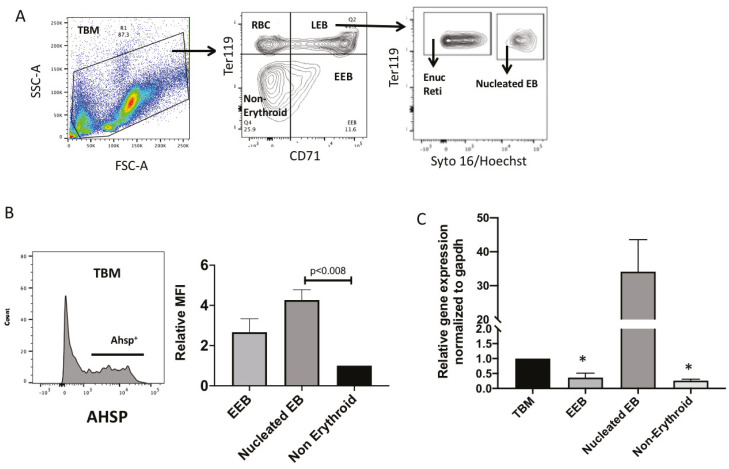
AHSP protein and gene expression from in vivo mouse bone marrow. (**A**) Exemplary flow cytometry gating of bone marrow, harvested from mice, stratified into total bone marrow (TBM), non-erythroid cells, early erythroblasts (EEB), red blood cells (RBC), late erythroblasts (LEB), enucleated reticulocytes (Enuc Reti) and nucleated erythroblasts (EB). (**B**) Intracellular AHSP protein expression of murine bone marrow in TBM, EEB, nucleated EB and non-erythroid subsets. (**C**) Relative gene expression of AHSP in bone marrow cells from flow sorted subsets as shown in A. Bar graphs represent fold change compared to TBM. Target gene cycle threshold (Ct) values were normalized to GAPDH Ct values using the ΔΔCt method. * *p* < 0.05 vs. TBM; *n* = 3.

The bone marrow is the site of homeostasis red blood cell production in mice and humans. Since mice reach adulthood between 8 and 10 weeks of age [9], we sacrificed mice when they were eight weeks old. To determine AHSP expression levels in an in vivo representation of red blood cell maturation we extracted total bone marrow cells from naïve healthy mice. We proceeded to analyze the total cell population using FACS analysis similar to our analysis of ex vivo cell cultures. We identified erythroblasts using CD71 on the *x* axis and Ter119 on the *y* axis (Figure 3A) as previously published [10]. Cells that express both CD71 and Ter119 are late erythroblasts (LEB) present in the top right quadrant. Those cells that only express CD71 and are yet to express Ter119 are early erythroblasts, also known as colony forming units (EEB or CFU-E) are located on the bottom right quadrant. Cells that express neither CD71 nor Ter119 are not developing red blood cells and are labeled as non-erythroid cells in the bottom left quadrant. In the top left quadrant are cells that only express Ter119 and are mature red blood cells that no longer express CD71. Immediate precursors to red blood cells are the LEBs, which can be further gated in terms of which LEBs have a nucleus present using the nuclear stains Syto16 or Hoechst. LEB cells that have a nucleus present are also called nucleated erythroblasts (NuEB). The other LEB cells that are further along in maturation and have extruded their nucleus are specifically named reticulocytes (Reti). In the histogram in panel B, total bone marrow cells show a subset of cells positively expressing AHSP. Comparing the mean fluorescence intensities between EEB + LEB versus nucleated erythroblasts confirms that nucleated erythroblasts have significantly higher fluorescence (*p* < 0.008). We then FACS sorted erythroblasts in various stages of red blood cell maturation and quantified AHSP gene expression with total bone marrow cells (TBM) as the base level. Results revealed the highest AHSP gene expression was found in nucleated LEBs likewise the to the protein expression. Furthermore, AHSP gene expression was significantly lower than TBM in EEBs and non-erythroid cells.

### 2.4. AHSP Expression of Ex Vivo Peripheral Blood Mononuclear Cell (PBMCs) Derived Erythroblasts

**Figure 4:**
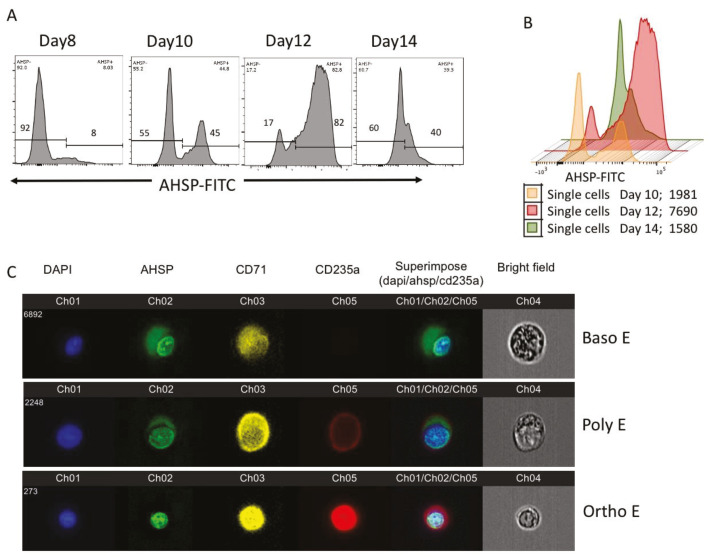
AHSP protein expression in human peripheral blood mononuclear cells. (**A**) Percentage of AHSP positive and negative cells in FACS analysis on culture days 8, 10, 12 and 14. (**B**) Overlay of AHSP protein expression displayed with color graphs on Day 10 (orange), Day 12 (red) and Day 14 (green) showing highest MFI shifted to right on Day 12. (**C**) Representative images from AMNIS image flow of AHSP expression in basophilic erythroblast (Baso E), polychromatophilic erythroblasts (Poly E) and orthochromatophilic (Ortho E) cell types. Cells were examined in all four fluorescent channels and brightfield. Blue stain is the nuclear stain DAPI, green is the AHSP marker, yellow is the transferrin marker CD71 and red is the glycophorin A marker CD235a. The superimposed image shows a combination of CD235a, AHSP and DAPI.

In addition to bone marrow stem cells, PBMC resident stem cells can also be used to develop red blood cells ex vivo [7]. PBMCs are advantageous to use in laboratory studies of erythropoiesis given that these cells are more readily obtained from human donors through venipuncture instead of a more invasive bone marrow aspiration from the soft tissue inside a donor’s bone. Results reveal that red blood cell maturation beginning with PBMCs took over two weeks of ex vivo cell culture, following a progression similar to bone marrow cells. The histograms in Figure 4A display percentages of AHSP positive cells over culture days 8, 10, 12 and 14. Expression of AHSP was first detected on day 8 with only 8% percent of total cells in culture expressing AHSP. The level of AHSP increased to 45% of cells on day 10 of cell culture and the peak AHSP protein expression of 82% on day 12 and then a decrease to only 40% of cells expressing AHSP on day 14. The overlay of AHSP mean fluorescence intensity expression shows that the day 12 histogram was furthest to the right on the *x* axis thus displaying the highest intensity of AHSP expression (Figure 4B; Day 10 = 1891, Day 12 = 7690 and Day 14 = 1580).

To assess various maturation stages of PBMC derived erythroblasts, we selected cultures from day 11 during which time we could find erythroblasts from early to late stages. Using Amnis, we imaged visual representations of basophilic erythroblasts, polychromatophilic erythroblasts and orthochromatophilic erythroblasts, the most mature at this time point respectively from the top row to the bottom row (Figure 4C). Comparable to bone marrow results, the least mature Baso E and Poly E cells expressed AHSP in the whole cytoplasm, whereas the most mature cells, the Ortho Es, only expressed green labeled AHSP colocalized with the blue labeled nucleus. 

### 2.5. Nuclear and Cytoplasmic Localization of AHSP in HuBM Derived Erythroblasts

**Figure 5:**
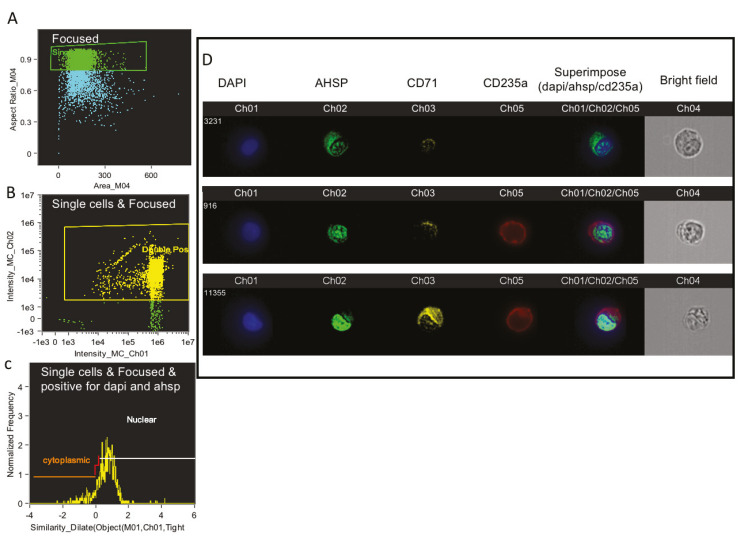
Representative images from AMNIS image flow of bone marrow derived erythroblasts on culture day 10 using multispectral imaging flow cytometry (MIFC) and analyzed by the IDEAS software. (**A**) Single cells were found on the camera’s focus. Then MIFC identified the nuclear and total cell areas using DAPI and brightfields respectively. (**B**) Dual expressing cells were gated on DAPI and FITC channel respectively. (**C**) Cytoplasmic area and nuclear area were defined on DAPI against frequency on histograms. The difference between the two were used to identify the nuclear and total cell areas using the DAPI and brightfield channels. (**D**) Representative images were selected to show the DAPI signal defining the nucleus and the remaining area within the overlaid brightfield image defining the cytoplasm to analyze RNA and protein expression and localization.

We used multispectral imaging flow cytometry (MIFC) to further confirm the expression and localization of AHSP. From day 11 culture, single cells were gated in the camera’s focus (Figure 5A) and the dual positive erythroblasts (AHSP^+^DAPI^+^) were then selected (Figure 5B) to identify the nuclear and total cell areas using the DAPI and brightfield channels, respectively. The IDEAS software then calculated the cytoplasmic area using the difference between the two as defined in Figure 5C, with the high nuclear-to-cytoplasmic ratios of most dual positive erythroblasts (AHSP^+^DAPI^+^). To measure AHSP translocation, we determined the ratio of Alexafluor 488 (FITC) signal in the nucleus versus the cytoplasm. For this analysis, the DAPI signal defined the nucleus and the remaining area within the overlaid brightfield image defined the cytoplasm. Figure 5D depicts representative images of AHSP expression both in the cytoplasm and the nucleus and in some erythroblasts congregated to the nucleus only. Having demonstrated that MIFC can be used to analyze RNA and protein expression and localization simultaneously, this technique can be applied in future experiments for comparative studies.

## 3. Discussion

We identified AHSP protein expression throughout erythropoiesis initially present in the cytoplasm and then culminating in the nucleus. With the use of imaging flow cytometry, we were able to provide evidence for the visual localization of AHSP throughout ex vivo red blood cell development. Remarkably, during erythroblast development using biphasic cell culturing, AHSP did not remain in its original localization, the cytoplasm. Instead, it began to translocate from the cytoplasm to the nucleus (on day 12) and eventually all of the AHSP was colocalized to the nucleus (on day 14). To our knowledge this is the first publication depicting AHSP protein expression from two different sources namely naïve bone marrow-derived and PBMC-derived erythroblasts. Moreover, we concurrently validated these ex vivo contrived observations in in vivo murine total bone marrow cells and confirmed that among nucleated erythroblasts, late erythroblasts expressed higher protein and gene expression for AHSP compared to early erythroblasts.

Examination of erythroblasts from bi phasic culture day 8 through culture day 14 illustrated in this manuscript was preceded by a preliminary analysis of HuBM obtained from four adult donor samples. Flow cytometry results depicting the percentage of AHSP^+^ cells (mean ± SEM) from culture day 9 and day 11 are shown in bar graphs in the Supplemental Figure S1. Based on this preliminary reproducible observation, we performed multiple techniques with samples from multiple culture days using the same starting donor HuBM for data representation defined in this manuscript.

Furthermore, surface antigens transferrin 1 (CD71) and glycophorin (CD235a) can be used to define the several erythroblast subtypes namely, CfuE, ProE, IntE (BasoE), PolyE, OrthoE and reticulocytes from day 9 and day 11 HuBM derived cultures [7,11] with slight modifications as in Supplemental Figure S2A,B. Based on the CD71/CD235a gating one can report the MFI of AHSP to inform subset based expression of the chaperone protein in the erythroblasts. Rather, we sought to image stream technology since the primary focus of this manuscript is to highlight the sub cellular localization of AHSP. Thus, we believe, just providing the MFI of AHSP from CD71/CD235a gating will not be sufficient. Additionally, we utilized multispectral imaging flow cytometry (MIFC) to further confirm the cytoplasmic and nuclear expression and localization of AHSP.

We identified that AHSP gene transcripts began to be expressed at low levels and then peaked with late maturation, declining to low levels in mature reticulocytes. Similarly, the translated protein progressively increased from early to late erythroblasts and peaks in nucleated BasoE, PolyE and OrthoE subsets, significantly decreasing during terminal maturation, tracking the same pattern as gene expression. This confirms the pattern of AHSP gene expression relayed by Dos Santos Oresco et al. 2002, though the specific peak of expression is two days later in our study, specifically day 12 and not day 8–10 range that Dos Santos Oresco reports [8]. This difference may be due to the later refined methodology of culturing PBMCs with serum free media and only adding cytokines specific to erythroblast differentiation. There was an additional effort to track AHSP gene expression in Varricchio et al. in 2010 but they did not identify a distinct decrease in late maturation stage of erythroblasts [12]. Indeed, they were unable to identify a statistical difference in the increase in AHSP mRNA perhaps due to, as they remark, the greater variability in their human blood donor samples.

In our current study, we observed erythroblast development periodically every other day from phase 2 of biphasic cultures from either human bone marrow or PBMC derived erythroblasts. The beginning of AHSP protein expression coincides with its mRNA expression on day 8 of culturing when AHSP transcription and protein was significantly detectable. Similarly, AHSP mRNA levels and AHSP protein levels peak at day 12 of ex vivo culturing. AHSP expression is so developmentally synchronized in the erythroid lineage that Yu et al., 2016 have proposed that expression of AHSP be used for the, “detection of erythroid elements within the bone marrow” [13]. Such a practice may miss early and later maturation stages of erythroid cells.

It is important to note that day 14 was also the beginning of the period of late maturation when the nucleus was ejected from the maturing reticulocyte. We were able to distinctly capture the process of ejection of a nucleus at this time point and can identify that AHSP localizes with the nucleus being ejected. Additionally, when we image mature condensed red blood cells without a nucleus there was also correspondingly an absence of AHSP. It is known that AHSP stabilizes newly formed *α*Hb effectively, preserving *α*Hb while preventing aggregation and reactive oxygen species mediated stress [2,14] by limiting *α*Hb reactivity through reversibly binding to the same location as βHb [15]. Therefore, we could interpret that excess free *α*Hb is sequestered by AHSP and translocated to the nucleus in maturing erythroblasts when effective hemoglobinization was accomplished.

This notion is further strengthened by probing AHSP negative cells on Day 8 and Day 14 based on nuclear staining by flow cytometry. These results reveal that during early stages of erythroid development, all of the erythroid progenitors were expressing nuclei, but only a few were expressing AHSP at low levels. Progressively by Day 12 about 2-fold more erythroblasts expressed AHSP. This then reversed back to very few cells expressing AHSP by Day 14 and the very same erythroblasts were devoid of nucleus. These results were reconfirmed by AMNIS image stream attesting nuclear localization during late OrthoE stage and eventually ejected with the nucleus in reticulocytes.

A recent applicable study on the quantification of the proteins in the extruded nucleus versus the cytoplasm of reticulocytes unfortunately did not mention AHSP [16]. Interestingly, hemoglobin subunit alpha, which is the binding partner of AHSP, was detected in both the nucleus and the cytoplasm [13]. Though at this juncture AHSP would have been outcompeted by hemoglobin beta in binding to hemoglobin alpha [15] functionally, it can be deduced that mature red blood cells need hemoglobin and not necessarily AHSP. The lack of AHSP in the reticulocytes may reflect its completed role in the developing erythroblast whereas hemoglobin remains essential for the red cell’s function. Autonomously in the arterial endothelium, free alpha hemoglobin is escorted to endothelial nitric oxide synthase (eNOS) by AHSP during nitric oxide degradation [17].

Therefore, the above study highlights free *α*Hb exclusive chaperone function of AHSP outside of red blood cells besides the crucial role of AHSP in stable hemoglobinization during erythroid maturation.

Nonetheless, in recognizing that AHSP is comparable to the heat shock family of proteins as members of chaperoning erythropoiesis proteins, Bell et al., 2013 identified that most heat shock proteins (hsp) are located in both the extruded nucleus and the mature reticulocyte except for hsp 90-alpha and hsp 4, which were mainly identified in the reticulocyte [3,16,18]. Though AHSP does not follow this pattern of localization, there were various other proteins, which were more localized with the extruded nucleus. The authors noted these proteins were related to the endoplasmic reticulum (ER). Bell et al. state that the majority of ER is extruded along with the nucleus. Previous research from the Weiss lab has identified the association of AHSP with ER and readily binding to the nascent transcribed AHSP to facilitate AHSP’s functional conformation [15]. Perhaps AHSP remains distinctly associated with the expansive ER thus being removed from the maturing red blood cell jointly with the ER. Future experiments are needed to clarify whether this is the case and may consist of colocalization assays with the erythroid ER.

## 4. Methods

### 4.1. Human Bone Marrow Cells

BM aspirate from an unidentified healthy male donor of 24 years age was obtained as a 1 mL excess portion from a clinical use of the main aspirate not related to this study. Samples were stored at −80 °C in 70% freezing media consisting of 90% fetal bovine serum with 10% dimethyl sulfoxide at 4 × 10^6^ cells per 1.5 mL cryotube (Sigma-Aldrich, St. Louis, MO, USA) until day of plating for cell culture.

### 4.2. Human Peripheral Blood Mononuclear Cells

Blood samples were collected from healthy adults over the age of 18 years enrolled as part of a larger related overarching study and were utilized towards this study. Institutional review board approval was obtained from the Loyola University Health Sciences division for use of human participants. Written informed consent to utilize samples for research purposes was obtained. Blood was drawn by a registered nurse in a purple vacutainer EDTA tube containing Ficoll-Hypaque (Bioscience, Franklin Lakes, NJ, USA). Within 30 min of blood draw, the vacutainer was centrifuged at 2900 rpm for 25 min at 22 °C to isolate PBMCs via gradient-density centrifugation. In 90% fetal bovine serum with 10% dimethyl sulfoxide (Sigma-Aldrich, St. Louis, MO, USA) 4 × 10^6^ PBMC cells were resuspended and stored at 80 °C until the day of culturing. Prior to plating, cells were assessed by Trypan blue exclusion for viability.

### 4.3. Cell Culture of Human Cells

Cells (bone marrow and PBMCs) were thawed in a 37 °C water bath for 1.5 min and then washed with Iscove’s Modified Dulbecco’s Medium (IMDM). The ACK (ammonium-chloride-potassium) lysing buffer was used to remove red blood cells from unfractionated bone marrow samples. Cells were then washed and placed in an expansion medium containing serum-free expansion medium (SFEM; Stem Cell Technologies, Vancouver, BC, Canada) supplemented with granulocyte macrophage colony-stimulating factor (20 ng/mL; eBioscience, San Diego, CA), stem cell factor (SCF; 45 ng/mL; Stem Cell Technologies) and interleukin-3 (20 ng/mL). In the last step, plasma from a healthy control study donor was added at 5% concentration per well, followed by incubation at 37 °C with 5% CO_2_.

On day 7, non-adherent cells were washed in IMDM and 0.05 × 10^6^ cells/well of a 12 well plate were seeded in SFEM supplemented with human recombinant Epo (1 U/mL; Stem Cell Technologies), holotransferrin (0.3 mg/mL; R&D Systems, Minneapolis, MN) and stem cell factor (10 ng/mL; Stem Cell Technologies) and 5% plasma from a control subject was added followed by incubation at 37 °C with 5% CO_2._

### 4.4. Fluorescence-Activated Cell Sorting (FACS)

On day 8, 10, 12, 14 and 16 time points, non-adherent cells were collected and washed in IMDM. Cells were stained with cell surface antibodies, anti CD235a cat# 551336 (BDbiosciences, San Jose, CA, USA) and anti CD71 cat# 555537 (BDbiosciences, San Jose, CA, USA) for 30 min at 4 °C. Next cells were fixed and permeabilized with 200 ul of Cytospin/Cytoperm (BD Biosciences San Jose, CA) followed by intracellular staining using anti AHSP cat# sc515436 (Santa Cruz Biotechnology, Dallas, TX, USA) for 2 h at room temperature, and then with Hoechst 33342 nuclear stain cat# H3570 (Thermofisher Invitrogen, Waltham, MA) for 10 min at room temperature. Cells were then analyzed by flow cytometry using BD LSRFortessa and gated with FlowJo software.

### 4.5. Amnis Single Cell Imaging

After 100,000 cells were analyzed per well using FACS, 10,000 of the remaining stained cells were run in the Amnis ImageStreamX MKII flow cytometer (Amnis Corporation, Seattle, WA, USA). For each individual cell the image of bright field view as well the fluorescence imagery of anti CD235a, anti CD71, anti AHSP and Hoechst nuclear stain were recorded at 60× magnification. Hoechst, FITC, PE and APC were excited with a 100 mW of 488 nm laser and 150 mW of 647 laser. Fluorescence was collected on channel one (430−505), channel two (488−505 nm), channel three (505−560 nm), channel five (560−595 nm) and channel four brightfield respectively. Ideas software (AMNIS Corporation) was utilized to analyze the images of anti CD235a in red, anti CD71 in yellow, anti AHSP in green and Hoechst nuclear stain in blue consistently using the same compensation matrix for each sample. Cells were gated on camera’s focus, followed by gating for the exclusion of debris. Next we gated for erythroblasts using anti CD71 and anti CD235a staining and finally graphed the intensity of fluorescence imagery of anti AHSP. The IDEAS software was used to overlay Hoechst nuclear stain, anti AHSP and anti CD235a.

### 4.6. Murine Animals for Bone Marrow

B6D2F1 mice weighing 25–30 g (Jackson Laboratories; Barr Harbor, ME, USA) were allowed to acclimatize for seven days following arrival at our Comparative Medicine Facility under a controlled temperature (20−22 °C) and humidity (20−40%) environment with a 12-h light—dark cycle. All experimental protocols were approved by the IACUC (Institutional Animal Care and Use Committee) of Loyola University Chicago Health Sciences Division.

### 4.7. Murine Bone Marrow Cell Extraction

Healthy naïve mice were sacrificed and on the same day bone marrow cells were harvested from the femurs of each mouse by flushing the marrow via a 25-guage needle through the proximal end of the femur and eluted into McCoy’s medium (Invitrogen, Carlsbad, CA, USA). Total bone marrow cells were then separated into subpopulations followed by gene expression analysis or staining for intracellular and surface erythropoietic cell markers (see details in separate sections below). For both procedures, murine bone marrow cells were stained with the following fluorochrome conjugated monoclonal antibodies specific for mouse: PE-CD71 C2 clone, cat # 553267 (BD Pharmingen, San Diego, CA, USA), PerCP.Cy5.5-Ter119 clone Ter-119; cat # 45-5921-82 (eBioscience, San Diego, CA, USA), Syto16—FITC; cat # S7578 (Fischer Scientific, Waltham, MA, USA) and CD16/CD43 Mouse BD FC Block clone 2.4G2 RUO; cat # 553141 (BD Pharmingen, San Diego, CA, USA), FITC-AHSP G-5 clone; cat # sc-515139-FITC (Santa Cruz Biotechnology, Dallas, TX, USA), Hoechst 33342 cat # H3570 (Thermofisher Invitrogen, Waltham, MA, USA) and OneComp eBeads; cat # 01-1111-41 (Invitrogen/Fischer Scientific, Waltham, MA, USA).

### 4.8. Murine Bone Marrow Cell Population Sorting

Briefly, 30 × 10^6^ cells total bone marrow (TBM) cells were labeled with the fluorochrome conjugated antibodies for 30 min at 4 °C in the dark. Cells were washed and resuspended in IMDM for sort purification as follows (FACS Aria). TBM cells were gated as non-erythroid (CD71^neg^ Ter119^neg^), RBCs (CD71^neg^ Ter119^pos^), early erythroblasts (CD71^pos^Ter119^neg^) and late erythroblasts (CD71^pos^ Ter119^pos^). Late erythroblasts were then separated into nucleated (Syto16^pos^) erythroblasts and reticulocytes (Syto16^neg^). About 50 × 10^4^ sorted cells from each subset were subjected to RNA extraction (See RT-qPCR methods section).

### 4.9. Murine Bone Marrow AHSP Staining

Briefly, 1 × 10^6^ cells total bone marrow (TBM) cells were labeled with the fluorochrome conjugated antibodies as mentioned in the previous section for 30 min at 4 °C in the dark. Cells were washed, fixed and permeabilized using Cytospin/Cytoperm (BD Biosciences San Jose, CA) following manufacture’s protocol followed by intracellular staining using anti AHSP for 2 h at room temperature. Hoechst nuclear stain was added for the last 10 min, washed and resuspended in PBS, and immediately analyzed by flow cytometry using BD LSR Fortessa and gated with FlowJo software. Positive and negative gates were set with FMO (fluorescent minus one) controls and unstained cells respectively. Flurochrome compensation was performed using OneComp eBeads.

### 4.10. Real Time Quantitative Polymerase-Chain Reaction (RT-qPCR)

Messenger ribonucleic acid (mRNA) was extracted from original bone marrow cells and PBMCs at various stages of erythropoietic maturation, day 8, day 10, day 12, day 14 and day 17 of cell culture, using the RNeasy kit (Qiagen, Hilden, Germany) as described by the manufacturer. RNA was transcribed into cDNA using the High-Capacity cDNA Reverse Transcription Kit (Applied Biosystems, Foster City, CA) and the Applied Biosystems Veriti 96-Well Fast Thermocycler 0.1 mL PCR machine. cDNA was appropriately diluted and combined in separate wells with three master mixes consisting of TaqMan Fast Advanced Master Mix (Applied Biosystems) and respective Taqman Gene Expression Assays (20X) consisting of either human AHSP:FAM cat #4448892 (Thermofisher Fischer Scientific, Waltham, MA), GAPDH:FAM cat #4453320 (Thermofisher Fischer Scientific, Waltham, MA, USA) and GATA-1:FAM cat #4453320 (Thermofisher Fischer Scientific, Waltham, MA, USA), or murine AHSP:FAM cat #4331182 (Thermofisher Fischer Scientific, Waltham, MA, USA), GATA1:FAM cat #4331182 (Thermofisher Fischer Scientific, Waltham, MA, USA) and GAPDH:VIC cat #4448489 (Thermofisher Fischer Scientific, Waltham, MA, USA) for respective samples. GAPDH was included as a normalization control for each analysis. The reaction took place in the StepOnePlus Real-Time PCR System (Applied Biosystems). Target gene cycle threshold (Ct) values were normalized to GAPDH Ct values using the ΔΔCt method. Results are expressed as fold change over day 8 BM derived erythroblasts’ gene expression.

## 5. Statistical Analysis

Results from murine cell sorting experiment are expressed as mean ± SEM. GraphPad Prism statistical program version 8.0 (San Diego, CA, USA) was used for the *t*-test analysis. Statistical significance was set at *p* < 0.05.

## 6. Conclusions

In conclusion, these experiments have revealed the previously unknown extrusion of AHSP in mature red blood cells. We were also able to outline AHSP protein expression and gene transcripts throughout ex vivo erythrocyte development from human bone marrow. Further, the same timing in cell cultures were noted whether starting with stem cells from peripheral blood or from human bone marrow, both peaking at day 12 of cell culture. In addition, we were able to confirm in an in vivo murine model of erythropoiesis that beginning expression of AHSP occurred in EEB and LEB with the highest amount of protein identified in the late erythroblast cells, which notably were not yet extruded their nucleus. Whereas in mature red blood cells or mature LEBs without a nucleus there was consistently an absence of AHSP. Future experiments are needed to determine the mechanism by which AHSP is ejected from the maturing erythrocyte whether through internalization in the extruded nucleus or extrusion with the closely associated ER.

## Supplementary Materials

The following are available online at https://www.mdpi.com/2409-9279/3/3/46/s1, Figure S1: Mean percentage of AHSP^+^cells. Figure S2: Exemplary gating strategy for erythroblast subset by the differential expressions of CD71 and CD235a.

## Abbreviations

AHSP	Alpha hemoglobin stabilizing protein
BasoE	Basophilic erythroblasts
EEB	Early erythroblasts
LEB	Late erythroblasts
MFI	Mean fluorescence intensity
NuEB	Nucleated erythroblasts
OrthoE	Orthochromatophilic erythroblasts
PolyE	Polychromatophilic erythroblasts
Reti	Reticulocytes
TBM	Total bone marrow cells
