# Supplementary material for: Stage Specific Expression Pattern of Alpha-Hemoglobin-Stabilizing-Protein (AHSP) Portrayed in Erythroblast Chronology
Source: Methods Protoc. 2020 Jun 30;3(3):46. doi: 10.3390/mps3030046 (PMC7563766; doi:10.3390/mps3030046)
Supplement: Supplementary file 1 [file mps-03-00046-s001.pdf]

Supplemental Figure 1

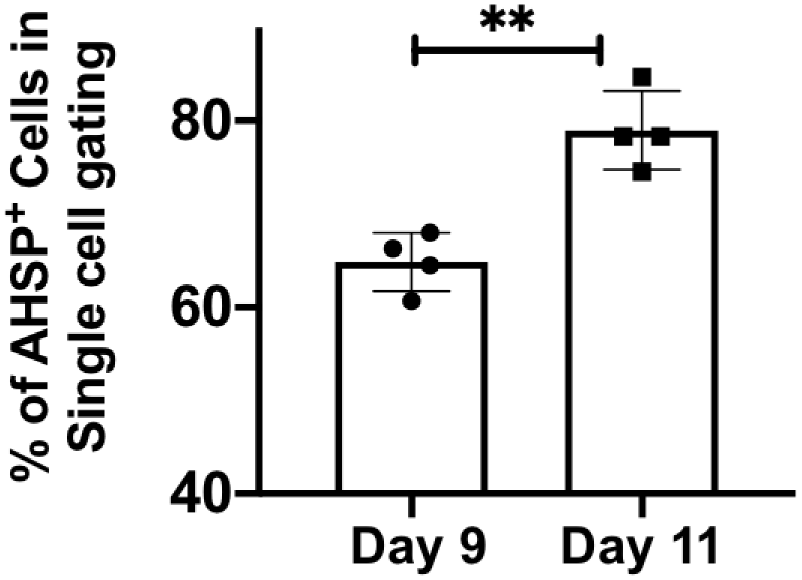

Supplemental Figure S1. Adult human donor bone marrow derived erythroblast cultures. Bar graphs represent Mean±SEM of the percentage of $AHSP^+$ cells gated for single cells from Day 9 and Day 11 culture respectively ($n = 4$).** $p < 0.002$ by unpaired two tailed t test with Welch's correction.

Supplemental Figure 2

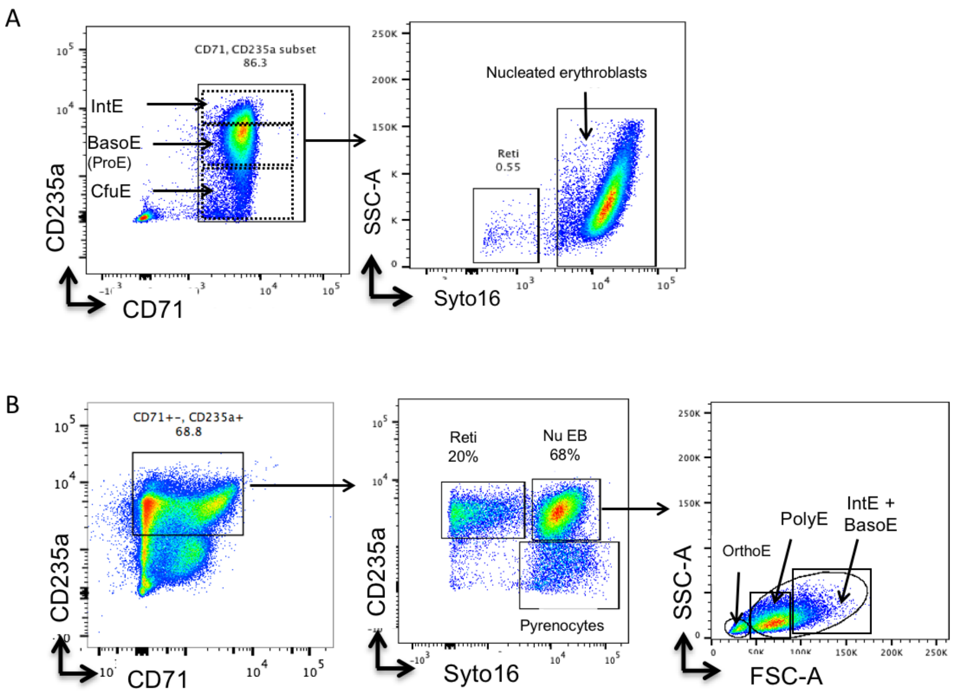

Supplemental Figure S2. Adult human donor bone marrow derived erythroblast cultures. (**A**) Day 9 cells gated out for live/dead, selected for single cells are viewed in scatter plots for CD71 in x-axis and CD235a in y-axis. Erythroblast subtypes with CD71^{hi}CD235a^{neg} are gated as Cfu-Es, those with CD71^{hi}CD235a^{med} are ProEs, and the ones expressing CD71^{hi}CD235a^{hi} are IntEs. (**B**) Day 11 cells gated out for live/dead, selected for single cells are viewed in scatter plots for CD71 in x-axis and CD235a in y-axis. CD71^{hi}CD235a^{hi} late erythroblasts are viewed in scatter plots for Syto16 (permeable nuclear dye) in x-axis and CD235a in y-axis. Those cells without the nucleus are reticulocytes (CD71^{hi}CD235a^{hi} Syto16^{neg}). The nucleated erythroblasts (CD71^{hi}CD235a^{hi} Syto16^{pos}) are stratified on the basis of size by plotting again on Forward and Side scatter. Representative gating for OrthoEs (SSC-A^{lo}FSC-A^{lo} , PolyEs (SSC-A^{med}FSC-A^{med}) and BasoEs + IntEs (SSC-A^{med}FSC-A^{hi}) are shown in the last panel.
